# Antibodies to leukotoxin A from the periodontal pathogen *Aggregatibacter actinomycetemcomitans* in patients at an increased risk of rheumatoid arthritis

**DOI:** 10.3389/fmed.2023.1176165

**Published:** 2023-08-03

**Authors:** Klara Martinsson, Andrea Di Matteo, Carina Öhman, Anders Johansson, Anna Svärd, Kulveer Mankia, Paul Emery, Alf Kastbom

**Affiliations:** ^1^Division of Inflammation and Infection, Department of Biomedical and Clinical Sciences, Linköping University, Linköping, Sweden; ^2^Leeds Musculoskeletal Biomedical Research Unit, LTHT and Leeds Institute of Rheumatic and Musculoskeletal Medicine, University of Leeds, Leeds, United Kingdom; ^3^Department of Odontology, Umeå University, Umeå, Sweden; ^4^Center for Clinical Research Dalarna, Uppsala University, Uppsala, Sweden; ^5^Department of Rheumatology, Linköping University Hospital, Linköping, Sweden

**Keywords:** ACPA, *Aggregatibacter actinomycetemcomitans*, rheumatoid arthritis, progression, periodontitis

## Abstract

**Objectives:**

Periodontitis and underlying bacteria have been linked to the development of rheumatoid arthritis (RA). One suggested pathogen is *Aggregatibacter actinomycetemcomitans* (*A.a*.), which expresses leukotoxin A (LtxA) that can citrullinate human proteins, providing a possible trigger for the production of anti-citrullinated protein antibodies (ACPA). In this study, we seek to determine the presence of antibodies toward LtxA in patients at risk of developing RA.

**Methods:**

Two prospective observational patient cohorts (one Swedish and one British) with symptomatic at-risk patients were studied. Anti-LtxA antibodies were analyzed by a cell-based neutralization assay in baseline serum and compared to 100 Swedish blood donors that served as controls.

**Results:**

Serum anti-LtxA levels or positivity did not differ between patients and blood donors. In the British cohort, anti-LtxA was more prevalent among ACPA-positive arthralgia patients compared with ACPA-negative arthralgia cases (24% vs. 13%, *p* < 0.0001). In the Swedish at-risk cohort, anti-LtxA positive patients were at increased risk of progression to arthritis (hazard ratio (HR) 2.10, 95% CI 1.04–4.20), but this was not confirmed in the UK at-risk cohort (HR 0.99, CI 0.60–1.65).

**Conclusion:**

Serum anti-LtxA is not elevated before RA diagnosis, and associations with disease progression and ACPA levels differ between populations. Other features of the oral microbiome should be explored in upcoming periodontitis-related RA research.

## Introduction

Connections between mucosal tissues and rheumatoid arthritis (RA) development have been attracting increasing interest in recent years (1). In this context, the oral cavity is of particular interest due to the reported association between periodontitis and RA (2). Such a link was further substantiated by the identification of protein citrullination properties of bacteria underlying periodontitis, providing a possible trigger for the production of anti-citrullinated protein antibodies (ACPA) (2, 3). *Aggregatibacter actinomycetemcomitans* (*A.a*.) is a Gram-negative coccobacillus that is recognized as a pathogen in periodontitis. Recent findings indicate high systemic immunoreactivity against *A.a*. when compared with several other bacterial species that are commonly detected in the human commensal microflora (4). *A.a*. produces a toxin called leukotoxin A (LtxA), which is a major virulence factor in periodontitis in young individuals (5). Intriguingly, LtxA was shown to trigger protein hypercitrullination in interaction with neutrophils, and antibodies to LtxA were increased in RA and correlated with ACPA levels (3, 6). These features of *A.a*. are of great interest considering that ACPA occurrence is a strong predictor of arthritis development among individuals with arthralgia (7) and of joint erosions in patients with recent-onset RA (8). Despite this, the role of *A.a*. in RA development and progression has been sparsely investigated. Nevertheless, it was recently shown that anti-LtxA IgM levels associate with RA onset (9), and circulating *A.a*. antibodies were associated with subclinical atherosclerosis in RA (10).

Previous studies on immune responses to *A.a*. have mostly used enzyme-linked immunoassays using *A.a*. LtxA as an antigen (3, 9). However, since the LtxA could be a starting point for the process leading to hypercitrullination and ACPA formation (3), it is of potential advantage to instead assess the functional (neutralizing) capacity of anti-LtxA in serum. The presence of systemic LtxA neutralizing activity has shown a strong correlation between systemic ab that binds LtxA (11) and the presence of A.a in the subgingival plaque (12). By using two cohorts of patients at risk of developing RA, we aimed to characterize the neutralizing anti-LtxA antibodies in relation to clinical course and autoantibody levels. This knowledge is of importance from an etiological perspective and to guide further work concerning dental interventions for the treatment of RA.

## Materials and methods

### Study populations

We included two independent at-risk cohorts, one Swedish with ACPA-positive patients only and one British with both ACPA-positive and ACPA-negative at-risk patients. Baseline characteristics are shown in Table 1. In addition, 100 Swedish healthy blood donors were included to serve as controls.

**Table 1:** Baseline characteristics of at-risk patients and controls.

	**TIRx**	**Leeds CCP positive**	**Leeds CCP negative**	**Controls**
	**(*****n*** = **82)**	**(*****n*** = **178)**	**(*****n*** = **86)**	**(*****n*** = **100)**
**Demographics:**				
Women, n (%)	66 (81)^a^	128 (72)^a^	65 (76)^a^	50 (50)
Age, mean (range)	51.8 (18–76)	51.3 (20–78)	52.0 (19–84)	51.7 (18–72)
Median follow-up time (IQR)	69 (24–90)	25 (10–53)		
Periodontitis (%)	-	72^b^	-	-
**Risk factors:**				
Ever smoker, n (%)	39 (48)	114 (64)^c^	-	-
Shared epitope carrier, n (%)	52 (64)^d^	90 (64)^e^	-	-
RF positive, n (%)	24 (29)	81 (46)^c, f^	-	-

^a^significantly different from controls *p* = <0.001, ^b^ *n* = 29, ^c^significantly different from TIRx patients *p* = <0.02, ^d^ n = 81, ^e^ n = 14 and ^f^ n = 177.

### At-risk patients

#### TIRx cohort

In the TIRx cohort (Swedish acronym for “xtra early rheumatology follow-up”), 82 patients with a positive IgG ACPA test in clinical routine and musculoskeletal pain of any sort and duration, but no baseline arthritis, were followed prospectively for the development of clinical arthritis (13). Patients were recruited between 2010 and 2013 at the rheumatology unit, Linköping University Hospital, Sweden. The exclusion criteria were previous rheumatic disease or treatment with oral corticosteroids within 6 weeks. Follow-up visits were scheduled regularly, and arthritis development was defined upon clinical examination by an experienced rheumatologist. The median follow-up time was 69 months [interquartile range (IQR) 24–90]. Progression to arthritis occurred in 39 out of 82 patients (48%) after the median 6 months (IQR: 1–71). A total of 15 patients (18%) had symptom duration up to 6 months, 27 (45%) patients had symptom duration between 6 and 18 months, and 30 (37%) patients had experienced symptoms >18 months prior to inclusion.

#### Leeds cohort

Anti-CCP positive at-risk individuals who took part in “The CCP Study: Coordinated Programme to Prevent Arthritis—Can We Identify Arthritis at a Pre-clinical Stage?” were enrolled from June 2008 to January 2019. A detailed description of the CCP study has been previously published (14, 15). In this national observational UK study, individuals who presented to their general practitioner (or other health professionals) with a new non-specific musculoskeletal symptom were tested for anti-CCP antibodies.

The individuals who have a positive anti-CCP2 test were then invited to Chapel Allerton Hospital, Leeds, UK, for further assessments at a dedicated research clinic as part of an observational longitudinal study until the development of inflammatory arthritis. Individuals with a negative anti-CCP antibody test were sent a postal questionnaire 12 months after enrollment, asking about their disease status (i.e., progression to inflammatory arthritis) (16).

For this study, we reviewed 178 anti-CCP positive individuals without baseline arthritis from the Leeds CCP cohort. Out of these subjects, 81 (46%) developed clinical arthritis during a median follow-up period of 25 months.

### Control groups

A total of 100 healthy blood donors recruited from Linköping University Hospital served as controls for the at-risk patient cohorts. These controls were age-matched to the TIRx cohort. In the UK, 86 anti-CCP negative subjects with arthralgia were selected as controls.

### Ethics

The ethical review board in Linköping, Sweden, approved the study protocol (Decision No. M220-09, 2015/236-32 and 2017/260-32). The Leeds CCP Study was approved by the NHS Health Research Authority National Research Ethics Service Committee Yorkshire and the Humber-Leeds West (REC reference: 06/Q1205/169). All participants signed written informed consent.

### LtxA antibody assay

Anti-LtxA antibodies were analyzed for their LtxA neutralizing capacity. This was detected as reduction in cell damage, assessed by neutral red leakage, following exposure to purified LtxA (17). THP-1 cells in RPMI-10% fetal bovine serum (FBS)-50 nM phorbol myristate acetate were seeded at 1 x 10^6^ cells/ml in flat 96 well plates and incubated at 37°C 5% CO_2_ overnight. The cells were washed with RPMI, and then patient serum and LtxA were added to the wells in triplicates. The plates were incubated for 2 h at 37°C with 5% CO_2_. The medium was removed, and 0.04 mg/ml neutral red diluted in RPMI-10% FBS was added to the wells and incubated for 90 min at 37°C with 5% CO_2_. The wells were washed with PBS (pH 7.4), and then 50% EtOH-1% acetic acid was added to lyse the cells. Following 10 min of incubation, the optical density (OD) was read at 650 nm (TECAN Sunrise, CA, USA). Anti-LtxA antibody neutralization capacity in percent was calculated by dividing serum sample OD with maximum cell viability OD (FBS) x 100. Serum samples inhibiting LtxA cell lysis >30% were classified as positive and <30% were classified as negative regarding anti-LtxA presence (11).

### Autoantibody analyses

Serum secretory component-containing (SC) ACPA and IgM ACPA were measured by modifying commercially available anti-CCP ELISA kits (Euro-Diagnostica, Malmö, Sweden) as described earlier (18). Serum IgA and IgG ACPA were analyzed by a fluoroenzyme immunoassay (EliA^TM^ Phadia AB, Thermo Fisher Scientific, Uppsala, Sweden) as described previously (18). RF tests were performed in a clinical routine setting at each local laboratory associated with the participating rheumatology unit. Free SC in serum was analyzed using an in-house sandwich ELISA as previously described (19).

### Statistics

Mann–Whitney U-test or Fisher's exact test was used to analyze differences between groups. The prognostic value of anti-LtxA antibodies was studied using Cox regression analysis and, when statistically significant in univariable analysis, adjusted in multivariable analysis for age, sex, smoking, RF status, ACPA levels, and symptom duration.

## Results

The baseline characteristics of patients and controls are shown in Table 1.

**Figure 1:**
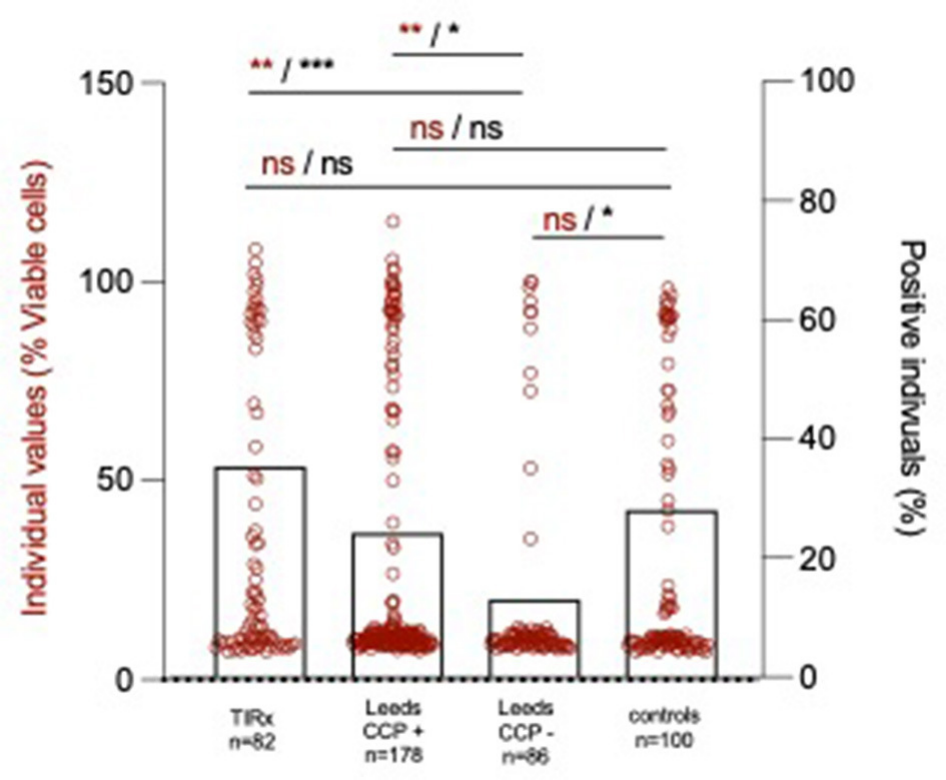
Individual anti-LtxA levels (red circles and statistical notations in red) and percentage positive individuals (black bars, mean ± SD and statistical notations in black). ^*^p < 0.05, ^**^p < 0.01, and ^***^p < 0.001.

There were no significant differences in anti-LtxA levels or percentage-positive individuals between the two at-risk cohorts and the controls (Figure 1).

### Serum anti- LtxA and arthritis development

**Figure 2:**
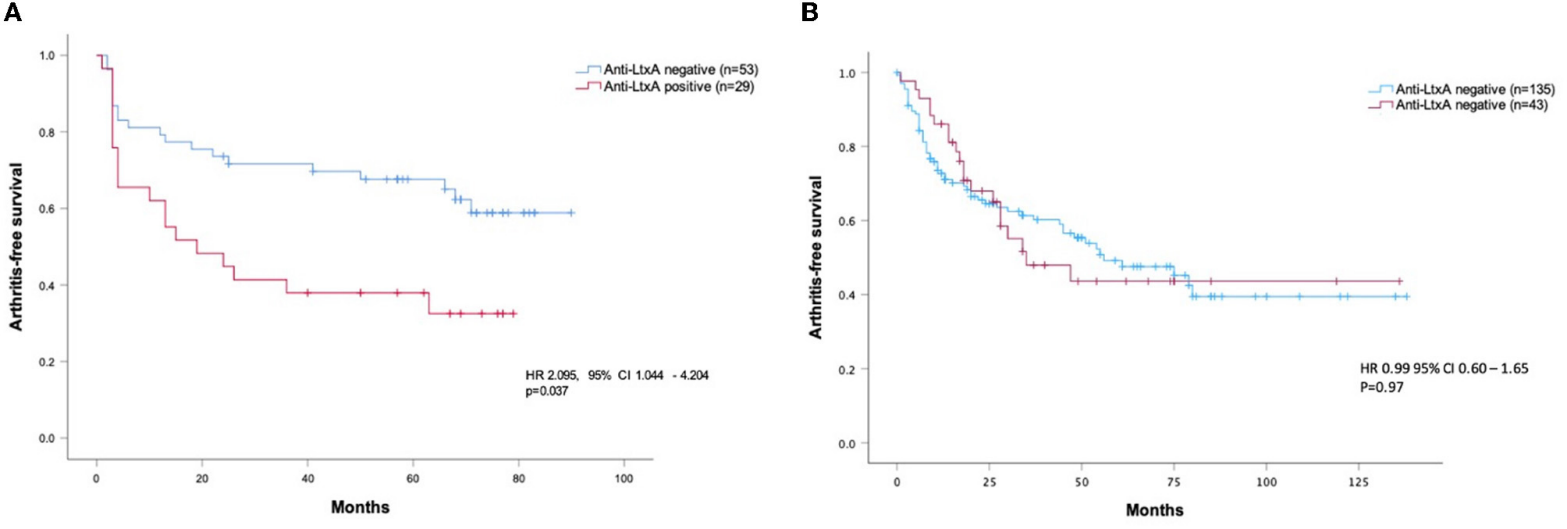
Baseline anti-LtxA levels vs. progression to arthritis in the TIRx cohort [**(A)** multivariable analysis] and the Leeds cohort [**(B)** univariable analysis]. Hazard ratio (HR) with 95% confidence interval (CI) in Cox regression adjusted for age, sex, smoking, RF status, ACPA levels, and symptom duration (TIRx only).

In the TIRx at-risk cohort, a larger proportion of patients progressing to arthritis was anti-LtxA antibody positive compared to those who did not progress (49% vs. 23 %, *p* = 0.021). This difference remained significant in univariable Cox regression analysis (HR 2.25, 95% CI 1.20–4.25 p = 0.012) and in multivariable Cox regression adjusted for possible confounders (HR 2.10, 95% CI 1.04–4.20, *p* = 0.037, Figure 2A). In the Leeds at-risk cohort, anti-LtxA seropositivity was not significantly different between progressors and non-progressors (25% vs. 24%, *p* = 1.0), and did not associate with arthritis development in Cox regression analysis (HR 0.99, CI 0.60–1.65, *p* = 0.973, Figure 2B).

### Serum anti-LtxA- and RA-related autoantibodies

Both the TIRx cohort and the ACPA-positive Leeds patients were more often anti-LtxA positive compared to the Leeds APCA-negative patients (35% vs. 13%, p = 0.001 and 24% vs. 13%, *p* = 0.035, respectively, Figure 1). Swedish blood donors (controls) were more often anti-LtxA positive than Leeds ACPA-negative at-risk patients (28% vs. 13%, *p* = 0.012, Figure 1). In TIRx, where all patients were IgG ACPA-positive, the levels were higher among patients positive for anti-LtxA compared with anti-LtxA negatives, with borderline statistical significance (mean 658 ± 1,007 vs. 334 ± 655 AU/mL; *p* = 0.073). A borderline significance was also seen in the Leeds cohort, where anti-LtxA positive showed higher IgG ACPA levels compared to anti-LtxA negative (mean 174 ± 149 vs. 133 ± 147 AU/mL, *p* = 0.068). Serum LtxA did not associate with IgA, IgM, SC ACPA, free SC, or RF status in the TIRx cohort (data not shown).

### Serum anti-LtxA and periodontitis

Data on periodontitis were present for a subgroup of the Leeds at-risk cohort, but anti-LtxA positive at-risk patients did not more often suffer from periodontitis compared to anti-LtxA negative at-risk patients (73% vs. 72%, *p* > 0.9).

## Discussion

To reach a better understanding of the possible role of *A.a*. in RA development and progression, we investigated toxin-neutralizing antibody responses in patients at risk of developing RA. The overall conclusion, from the present and previous studies (3, 9), is that *A.a*.-related antibody responses, as well as possible links to RA, appear to be substantially influenced by cohort characteristics and/or the context from which they have been recruited. In the Swedish at-risk cohort, we found neutralizing antibodies to *A.a*. LtxA prognostic for arthritis development among symptomatic ACPA-positive patients also after adjustments for smoking and other possible confounders. However, this association could not be replicated in the UK at-risk cohort despite inclusion criteria and baseline characteristics being very similar. In both at-risk cohorts, there were indications that a humoral response to LtxA associates with ACPA production, which is in agreement with the seminal study in established RA by Konig et al. (3). However, at-risk patients (ACPA-positive) did not have elevated anti-LtxA compared to (ACPA-negative) blood donors, possibly implying that *A.a*. enhances rather than initiates an ACPA response.

A major strength of this study is the inclusion of patients prior to arthritis onset, which enables proper determination of whether *A.a*. immunity precedes RA development or not. The use of two independent and geographically distinct cohorts, which resulted in discordant results, highlights that anti-LtxA results cannot readily be extrapolated across populations. There was a slightly lower occurrence of anti-LtxA in the British population compared to the Swedish, but whether or not that reflected a lower prevalence of *A.a*. or periodontitis could not be investigated in this study. Nevertheless, previous studies show a prevalence of periodontitis of 38% in the UK (20) and 40% in Sweden (21) suggesting that the prevalence of periodontitis does not stand for the slight differences in anti-LtxA ab occurrence between the two at-risk cohorts. Another possible drawback is that we did not specifically address antibody isotypes but instead applied a functional assay to detect anti-LtxA. We believe, however, that given the prevailing hypothesis of LtxA-induced hypercitrullination as a mechanistic link to RA, the total neutralizing capacity of the anti-LtxA antibody response is a relevant readout. We did not examine *A.a*. presence in the oral cavity, but it was previously reported from the UK at-risk cohort that *A.a*. DNA abundance was neither increased nor prognostic for arthritis development (20).

To conclude, we found some associations between *A.a*. and different aspects of RA development and progression. However, the results are not consistent across populations, implying that other features of the oral microbiome should be explored in upcoming periodontitis-related RA research.

## Data availability statement

The raw data supporting the conclusions of this article will be made available by the authors, without undue reservation.

## Ethics statement

The studies involving human participants were reviewed and approved by the ethical review board in Linköping, Sweden (Decision No. M220-09, 2017/260-32) and NHS Health Research Authority National Research Ethics Service Committee Yorkshire and the Humber—Leeds West, UK (REC reference: 06/Q1205/169). The patients/participants provided their written informed consent to participate in this study.

## Author contributions

AK and AJ conceived and planned the experiments. KMar, CÖ, and AD carried out the experiments. AK and KMar took the lead in writing the manuscript. All authors contributed to the interpretation of the results, provided critical feedback, helped shape the research, analysis, and manuscript.
